# Supplementary material for: Learning curve and surgical outcome of robotic assisted colorectal surgery with ERAS program
Source: Sci Rep. 2022 Nov 29;12:20566. doi: 10.1038/s41598-022-24665-w (PMC9709162; doi:10.1038/s41598-022-24665-w)
Supplement: Supplementary file 1 — Supplementary Information 1. [file 41598_2022_24665_MOESM1_ESM.zip › LSC Raw data-Ñ[▒K/149/O_ñw╝╨░O▒Kñσ.pdf]

正式摘要(電子簽章：000000233752417)

請選擇...reporter

臺中榮民總醫院

Operation Note

[redacted]

- 手術日期：2022/01/06
- 手術醫師：蔣鋒帆，VS 江世偉，Fellow 楊曙榮，R 蔡濬樟，R 林志安
- Anesthesia：GE
- Position：Supine
- Aseptic agents：Soap，Betadine Alcohol，Betadine Aqua

Diagnosis

- Pre-operative diagnosis : RO Malignant tumor of A-colon
- Post-operative diagnosis : Ascending colon leision suspect benign leision s/p laparoscopic right hemicolectomy

Surgical Indication

- Removal of tumor

Operation

- Laparoscopic right colectomy and anastomosis

Finding

- 1. skip leision from cecum to hepatic flexure with fibrotic band in A-colon
  2. No liver metastasis and no peritoneal seeding
  3. Side to side anastomosis with GIA 75/3.8 with two fires.
  4. Ligation of ileocolic, and right branch of middle colic vessels.
  5. No drain, Surgiwrap:+

Procedure

- Under general anesthesia, the patient was placed in the supine position with legs in adducted position. The abdomen was prepped and draped in standard sterile fashion. A small vertical incision was made below the umbilicus and a 12mm trocar was inserted into the peritoneal cavity by open method. Pneumoperitoneum was established with CO2 inflation. Laparoscope was inserted and then a 12mm trocar were inserted over LUQ of abdomen. Two 5mm trocars were inserted over RLQ and LLQ of abdomen. The liver, omentum and peritoneum were inspected for evidence of metastatic disease. No metastatic disease was noted. The peritoneum overlying the mesentery was then scored with electrocautery and the ileocolic artery was identified, ligated with endoclips, and transected. The remaining vessels to the cecum were also divided and ligated. Using Harmonica, omentum was freed from the transverse colon and lesser sac was entered. The colon was freed from its peritoneal attachments along the line of Toldt from the cecum to the hepatic flexure. Hemostasis was checked in the operative field. Cecum, ascending colon and hepatic flexure were mobilized and then laparoscope was removed. One midline incision was made at peri-umbilical region. The ascending colon was delivered into the incision and points of transection were selected proximally and distally. Side to side anastomosis was performed with endo GIA 75/3.8. Interrupted 3-0 silk Lambert sutures were used to re-inforcement at outer layer. The anastomosis was checked and found to be intact and widely patent. The fascia was closed with interrupted sutures of No.1 Vicryl. The fascia of the 12 mm trochar wound was closed with 1-0 vicryl. The skin was closed with 3-0 Eutek and Surgiseal. The patient tolerated the procedure well and was brought to the POR in stable condition.
- Estimated blood loss: minimum
- 器械數量: 正確
- 紗布數量: 正確

Image

- PAINT
  - abdomen

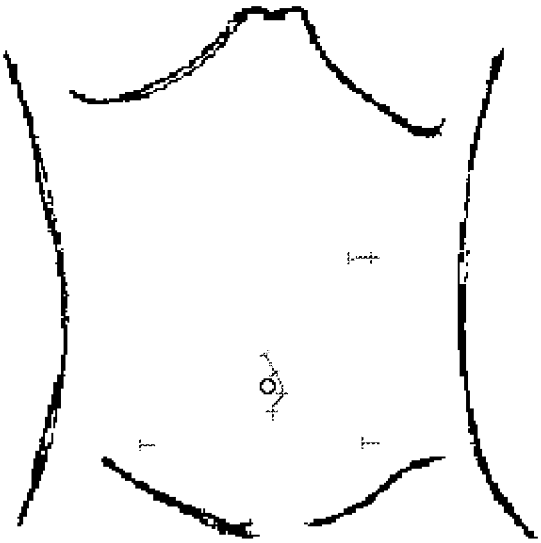

  - colon & rectum

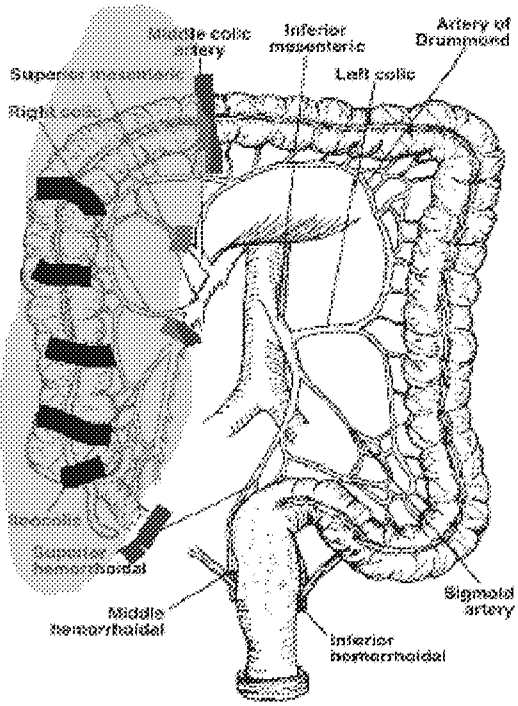

主任 2062F蔣鋒帆 / 醫師 2292G楊曙榮

正式摘要(電子簽章：000000233752417)

請選擇...reporter

doctorRank:B
